# Sensitivity and spectral control of network lasers

**DOI:** 10.1038/s41467-022-34073-3

**Published:** 2022-10-30

**Authors:** Dhruv Saxena, Alexis Arnaudon, Oscar Cipolato, Michele Gaio, Alain Quentel, Sophia Yaliraki, Dario Pisignano, Andrea Camposeo, Mauricio Barahona, Riccardo Sapienza

**Affiliations:** 1 The Blackett Laboratory, Department of Physics, Imperial College London, London, UK; 2 Department of Mathematics, Imperial College London, London, UK; 3 Blue Brain Project, École Polytechnique Fédérale de Lausanne (EPFL), Campus Biotech, Geneva, Switzerland; 4 Department of Chemistry, Imperial College London, London, UK; 5 NEST, Istituto Nanoscienze-CNR and Scuola Normale Superiore, Pisa, Italy; 6 Dipartimento di Fisica, Università di Pisa, Pisa, Italy

**Keywords:** Lasers, LEDs and light sources, Photonic devices, Lasers, LEDs and light sources, Metamaterials

## Abstract

Recently, random lasing in complex networks has shown efficient lasing over more than 50 localised modes, promoted by multiple scattering over the underlying graph. If controlled, these network lasers can lead to fast-switching multifunctional light sources with synthesised spectrum. Here, we observe both in experiment and theory high sensitivity of the network laser spectrum to the spatial shape of the pump profile, with some modes for example increasing in intensity by 280% when switching off 7% of the pump beam. We solve the nonlinear equations within the steady state ab-initio laser theory (SALT) approximation over a graph and we show selective lasing of around 90% of the strongest intensity modes, effectively programming the spectrum of the lasing networks. In our experiments with polymer networks, this high sensitivity enables control of the lasing spectrum through non-uniform pump patterns. We propose the underlying complexity of the network modes as the key element behind efficient spectral control opening the way for the development of optical devices with wide impact for on-chip photonics for communication, sensing, and computation.

## Abstract (web summary)

Nanophotonic light sources with programmable emission spectrum are important building blocks for integrated photonics, sensing and optical computing. Here the authors tune the complex laser spectrum of a network laser achieving selective lasing of a single, two or more modes.

## Introduction

Lasers with a well defined emission frequency and direction have revolutionised many fields, from material processing to biophysics and communication, just to mention a few. Traditionally, the spectral properties of the laser are inherited directly from the modes of the passive cavity, and mode selection achieved by engineering spectral feedback or absorption. The laser is usually designed to suppress multimode lasing and favour single-mode operation. In contrast, random lasers are an unconventional lasing architecture where light is amplified in a multimode scattering medium, thus supporting many lasing modes at random frequencies^1–3^. The ensuing low-coherence, multi-frequency, fluctuating laser radiation has applications in low-coherence imaging^4^ and super-resolution spectroscopy^5^, but is not suited for technologies that require precise control over the lasing modes, such as sensing^6^, communication^7^ or optical computing^8^. An experimental challenge is therefore how to achieve spectral selection in a controlled manner from such random lasing architectures. Towards this goal, pump engineering has been explored to obtain spectral selection in powder random lasers^9^, in deformed microdisk cavities^10^, and in a one-dimensional opto-fluidic random laser^11^.

Recently, a novel type of random laser called network laser was introduced in Refs. 12, 13. Network lasers consist of active single-mode waveguides connected according to a network topology. The passive modes of such systems are captured by quantum graphs^12^ and scattering matrix models^13^. Yet to take into consideration mode competition and nonlinear interactions, one must go beyond such passive models and solve the Maxwell-Bloch equation^14^ or its steady state ab-initio laser theory (SALT) approximation^15^ on a graph. This leads to a problem in nonlinear quantum graphs, recently studied in the context of the nonlinear Schrödinger equation^16,17^, but not yet considered to formulate the spectral control of network lasers.

Beyond photonic systems, how to design network structures or their inputs to produce specific dynamic behaviours is a central question in many areas, such as in the haemodynamics of arterial networks^18^, power grids^19^, brain networks^20^, or acoustic waves in elastic networks^21^. In conventional networks^22^, simple graph-theoretical measures are often sufficient to controllably characterise and produce network outputs^23^. However, such simple network measures are rendered unsatisfactory in nonlinear quantum graphs due to the complex interplay between graph structure and dynamical processes^24^.

Here we show that the underlying complexity of the nonlinear quantum graphs associated with random lasing can be harnessed to achieve a high degree of design control on the lasing emissions. We demonstrate experimentally and numerically that the complex emission spectrum of nanophotonic network lasers can be efficiently and precisely controlled through optimisation of spatially non-uniform pump patterns.

## Results

### Network laser spectral sensitivity

**Fig. 1:**
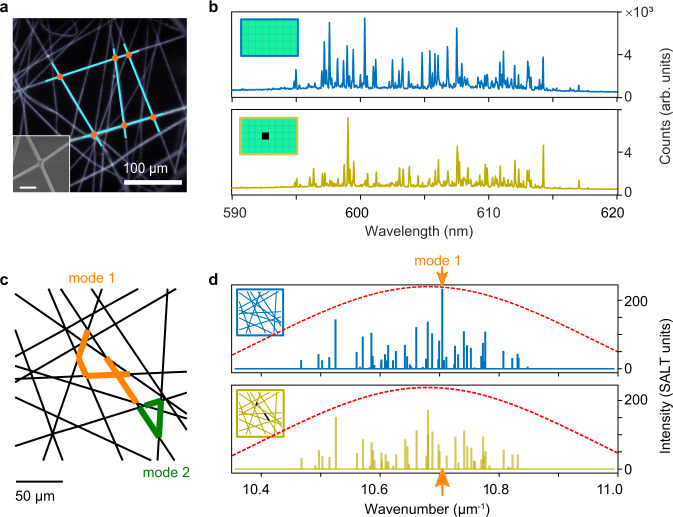
Spectral sensitivity of network lasers to pump profile. **a** Fluorescence image of a photonic network with interconnected dye-doped polymer nanofibers. As a guide to the eye, we highlight the graph topology (edges as blue lines, nodes as orange dots) over a few links as an example. Inset: scanning electron microscopy image of a node of the network formed by annealing two nanofibers (scale bar = 1 *μ*m). The nanofibers have diameters in the range 200–500 nm and function as single-mode waveguides for light emitted by the dye. **b** Lasing spectrum of the network in **a** obtained with uniform illumination (blue) and with a slightly modified pump pattern (yellow), both at pump fluence of 1.5 mJ cm^−2^ pulse^−1^. The insets show the respective pump patterns; the illumination area on sample is 300 × 480 *μ*m^2^ and the modified pattern has pump removed from a small area 60 × 60 *μ*m^2^ in the centre of the illuminated rectangle. **c** A model planar photonic network modelled as a Buffon graph that is open at the boundaries. We highlight edges with more than 50% of the maximal amplitude of the electric field for a delocalised mode (orange edges, mode 1) and a localised mode (green edges, mode 2). **d** Numerical calculations of the lasing spectrum from the Buffon network in **c** obtained with netSALT. Spectra at pump power *D*_0_ = 0.01 (SALT units) show ~ 50 lasing modes within the gain spectrum of dye (red dashed line). Note the suppression of mode 1 when changing from uniform pumping (blue, pump profile in inset) to a pump missing the two edges supporting the largest electric field amplitudes for mode 1 (yellow, pump profile in inset).

The network lasers examined here are planar and built from dye-doped polymer nanofibers physically joined together at the nodes^12^, resulting in graph-like structures with an average node degree of 4 and edge lengths ranging from 10 to 100 *μ*m (Fig. 1a). Lasing is experimentally obtained by optical pumping using a custom-built lasing microscope (see Methods). When uniformly pumped over a 300 × 480 *μ*m^2^ rectangular area, the networks lase from multiple modes, with narrow linewidths ( ~ 50 pm), as shown in Fig. 1b and Supplementary Fig. 1. These modes are formed by interference of light over multiple closed loops in the network and amplified by optical gain in the network links. Typically, 30 to 100 lasing modes are observed within the gain bandwidth of the dye.

The lasing spectrum is highly sensitive to changes in the spatial profile of the optical excitation. When the experimental pump pattern is modified by removing a small central area of 60 × 60 *μ*m^2^ from the illuminated region, (corresponding to a 7% reduction of the net pump energy delivered to the sample), we observe a drastic change in the lasing spectrum, which is stable when illuminated repeatedly with the same pump pattern (Fig. 1b). Some modes are amplified (up to 280%) while others are attenuated (down to 20%), and even new modes (not lasing under the uniform pump) lase (see Supplementary Fig. 2).

To understand the sensitivity of the network laser to non-homogeneous pump profiles, we developed a numerical model, called netSALT, which solves the nonlinear interaction of the optical waves on networks within the SALT approximation^15^ (see Methods and Supplementary Notes 1-3 for full details). The netSALT model includes amplification and loss on graph edges and mode competition. Under uniform pumping, the predicted spectrum in Fig. 1d is qualitatively similar to the experimental one, with similar number of modes (see Supplementary Fig. 3c).

The high sensitivity arises because of a large number of modes competing for gain, some delocalised and other localised, as shown in Fig. 1c and Supplementary Fig. 4. The network modes are spatially coupled as they partially overlap on graph edges; in this particular graph, there are 450 modes within a spectral range of 35 nm. If we select the mode with highest modal intensity (mode 1 in Fig. 1d) and turn off the pump illumination from the two edges supporting the largest electric field amplitudes for this mode (pump profile shown in inset), mode 1 does not lase anymore and overall most lasing modes change in intensity (see Fig. 1d and Supplementary Fig. 3a).

### Processes involved in mode selection

**Fig. 2:**
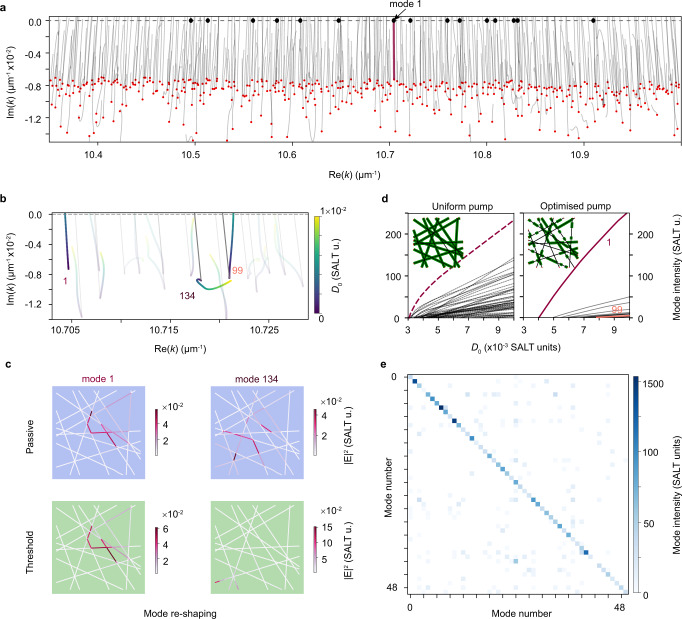
Achieving single mode lasing through non-uniform pumping (theory). **a-b** NetSALT calculations for lasing on a Buffon graph with non-uniform pumping indicate various processes for mode selection. **a** Mode trajectories in the complex *k* plane (shown over large Re(*k*) range, with red dots identifying the passive modes and black dots identifying the modes that lase. The non-uniform pump profile used to obtain these trajectories is shown in **d**, right-hand panel inset. **b** Zoom of **a** over a small range of wavelengths with trajectories shown in colour, where the colour scale indicates the pump strength. Three modes (1, 99, and 134) are highlighted to exemplify the different processes involved in mode selection. Mode trajectories with uniform pumping are shown by solid grey lines. **c** Example mode profiles for two network modes (1 and 134) before pumping (passive, *D*_0_ = 0) and at threshold for mode 1 (*D*_0_ = 0.004) and at pump strength *D*_0_ = 0.01 for mode 134. Minimal change in profile is observed for mode 1, whereas mode 134 reshapes significantly. **d** Modal intensities as a function of pump power for a uniform pump and a pump chosen to maximise lasing of mode 1 (see Methods), with pump profiles represented as green edges in insets. **e** Heatmap of the modal intensities of the first 50 modes (along each row) under 50 patterns optimised for each mode (each column). The computations correspond to the top 50 modes of the Buffon graph ordered in descending order of *Q*-factor. Optimisation of the pump profile leads to good mode selectivity.

The high sensitivity of the network laser to pump illumination can be used for designing a pump to either select or suppress lasing from certain modes. The main underlying processes that determine the lasing spectrum under a non-uniform pump are: a) efficient pumping of a mode to reach threshold at lowest pump power compared to all other modes; b) mode reshaping; and c) mode competition. To illustrate these processes, we use netSALT to calculate the lasing modes of a complex network when illuminated with a non-uniform pump profile with 50% fill fraction (shown in inset of right panel in Fig. 2d), chosen to maximise the modal intensity for mode 1. As shown in Fig. 2a, the network has 454 passive modes (red dots) in the displayed region of the complex wavenumber *k* centred around the gain curve. Of these, only 208 modes reach lasing threshold ($|{{{{{{{\rm{Im}}}}}}}}(k) |=0$) when pumped with a pump strength of up to *D*_0_ = 0.01 with the given non-uniform pump profile. Of these 208 modes that can potentially lase, only 15 modes (black filled circles) lase due to strong mode competition captured by the netSALT calculation.

Figure 2b is a zoomed view of the complex *k* plane, highlighting three modes, labelled 1, 99, and 134 (sorted by *Q*-factor) and their trajectories for uniform (grey line) and non-uniform (coloured line) illumination (see also Supplementary Fig. 5). These trajectories are obtained by tracking the modes under increasing *D*_0_ (with increments 1.4 × 10^−4^). Some modes (e.g. mode 1) move directly and rapidly towards the lasing threshold under the increase of *D*_0_, while others (e.g. mode 99) undergo nonlinear shifts in resonance frequencies and thus reach lasing threshold at higher values of *D*_0_. In other cases, modes (e.g. mode 134) can move away from the lasing threshold and never reach it within our range of pump power. This behaviour is due to the second process, i.e. mode reshaping, which changes the mode amplitude on each edge and therefore modifies the condition for resonance. In extreme cases, such as for mode 134 (see Fig. 2c) which spatially shifts towards the network boundary, the trajectory in the *k* plane stalls. Lastly, mode competition, which is the nonlinear interaction for gain above threshold due to spatial hole burning, affects the modal intensity of competing modes as well as their effective lasing thresholds, also called interacting lasing thresholds^15^. Mode competition depends on many factors, including spatial overlap between modes, mode frequencies with respect to the gain spectrum and pump power required to reach threshold, and is thus also dependent upon the pumping efficiency and mode reshaping.

### Theoretical modal control

The complex modal interaction and these three processes can be exploited to achieve mode selection by adaptive pumping. We give one example in Fig. 2d by comparing a uniform pump against a pump optimised to lase mode 1 (whose mode profile is shown in Fig. 2c). The light-in light-out (LL) curves (or modal intensities as a function of pump power) show clear improvement in the mode suppression ratio for mode 1 with optimised pumping. Even if other modes lase at higher pump power this mode has an intensity significantly higher than all other modes across the power range. Notice that mode 1 lases first, with a large gap of lasing threshold with the next lasing mode (see Supplementary Fig. 6).

In general, finding the right illumination pattern to achieve a desired lasing spectrum, e.g. single mode operation, is not a trivial task (see Methods for a description of the optimisation). Furthermore, to find pump patterns that are physically relevant, experimental limitations on the pump spatial resolution, pump power and optical gain have to be considered. We note that the naive approach of pumping the edges where the target mode has a large electric field does not always ensure single mode lasing, in particular for modes that are spatially delocalised or have high losses (see Supplementary Fig. 7). Instead, with optimised pump profiles, we can lase 143 out of the 200 modes, with a side mode suppression ratio larger than one, and lase 102 with a suppression ratio larger than two. The matrix in Fig. 2e shows in each row the modal intensities of the optimised pumping of the first 50 modes. These modes are arranged in the matrix in order of *Q*-factor, where mode 0 has the highest *Q*-factor. A large value on the diagonal corresponds to a good performance single mode lasing and low values on the off-diagonal indicates strong suppression of the unwanted lasing modes. We observe that control can be achieved across a large frequency window, even far from the gain maximum, as well as for relatively lossy modes (see Supplementary Fig. 7). After pump optimisation, 90% of the top 50 modes (and 70% of the top 200 modes) can be controlled with intensity more than double than any other mode. We remark that most of the obtained optimal pump profiles have only a partial correspondence to the target mode profiles.

### Experimental demonstration of spectral control

**Fig. 3:**
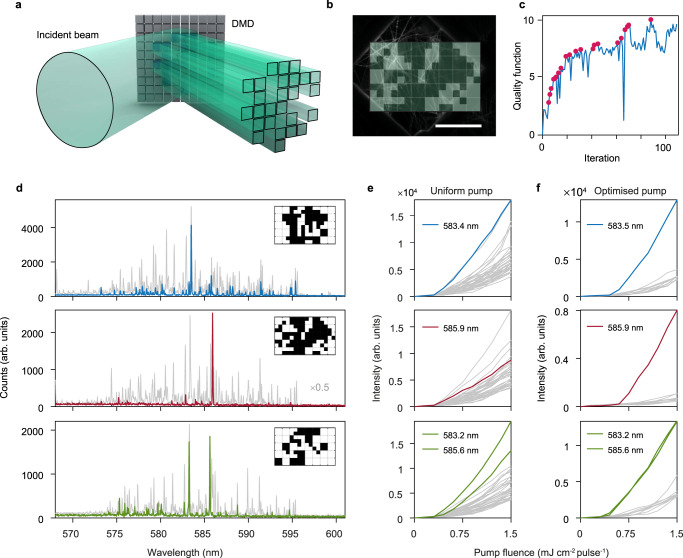
Experimental spectral control of network lasers. **a**, **b** Lasing modes in the network laser are controlled by selective illumination of the individual network links. This is done by shaping the pump laser using a digital micromirror device (DMD). The patterns are projected on the sample and cover a rectangular area of 300 × 480 *μ*m^2^ (scale bar = 200 *μ*m). **c** Plot of the quality function at each iteration for the optimisation of mode at 585.9 nm (shown in red in **d–f**). The pump pattern is optimised using a derivative-free, greedy iterative algorithm (see Methods) that optimises the quality function Equation 1 to improve the mode suppression ratio, leading to a progressive suppression of the unwanted modes while the selected one is maintained. Pink dots indicate improvements in the quality function during the optimisation. **d** Lasing is controlled from a multimode spectrum (grey) for homogeneous pumping to a single mode, shown here for two examples at 583 nm (blue) and 586 nm (red), obtained when illuminating with the patterns shown in insets. Bi-modal lasing, i.e. enhancement of both modes at 583 and 586 nm (green) is also achieved under a different illumination pattern. Fill fraction of the optimised patterns are 0.57, 0.46, 0.68, for the blue, red, and green spectra, respectively. Optimisation is performed at pump fluence of 1 mJ cm^−2^ pulse^−1^ for all three spectra shown, which is ~ 3 × larger than the lasing threshold when uniformly pumped. The evolution of the emitted intensity as a function of pump fluence when pumping with the uniform and optimised pump patterns are shown in **e** and **f**, respectively.

The high mode selectivity of network lasers predicted through numerical calculations is observed experimentally. Following the approach of Refs. ^9,11,25,26^, we use a digital micromirror device (DMD) to project different pump patterns on the sample (Fig. 3a, b), discretised into binary intensity pixels. Limitations on the spatial resolution of the pump, maximum amount of power available for pumping and the amount of gain in the medium constrain the parameter space to find physically relevant pump patterns. Patterns are optimised using a derivative-free, greedy iterative algorithm (see Methods). The mode suppression ratio between the target mode and other lasing modes is computed at each iteration to form a quality function to be maximised (see Methods and Fig. 3c). The results of such optimisations for the first and fourth largest modes, and the initial spectrum under uniform pumping (grey), are presented in the top and middle panels of Fig. 3d. The LL curves under uniform and optimised pumps (Fig. 3e and f, respectively) shows a successful suppression of undesired lasing modes, while maintaining the intensity of the target mode. Additional results of single mode lasing optimisation from different areas of the sample and at larger pump power are given in Supplementary Figs. 8-9. Furthermore, we experimentally demonstrate that it is possible to optimise for concurrent lasing of several modes, as shown for two modes in the bottom panels of Fig. 3d–f. We numerically confirmed this result in Supplementary Fig. 10, and assess the experimental stability of the spectra by switching repeatedly between different pumps in Supplementary Fig. 9.

## Discussion

Our procedure for single-mode optimisation converges in ~ 100 steps, both in experiments and theory, while exploring a configuration space of 2^160^ configurations (for a 16 × 10 pixels discretisation). This remarkable efficiency indicates the existence of correlations emerging from the underlying physical constrain of light waves on a network, whose modes are not random and discontinuous, but localised and continuous.

Underlying all the physical processes that occur in a network laser when optically pumped, the network and its specific topology dictates the form of the lasing spectrum. The network topology defines the mode density, their *Q*-factor and spatial distribution, which affects controllability of the lasing spectrum. For example, as an extreme case, simple graphs such as rings have modes with large spatial overlaps (see Supplementary Fig. 4), with only those in the centre of the gain spectrum that can be controlled. In contrast, large planar networks will contain many modes over a wide range of localisations with many possible single lasing regimes. The large controllability of our network lasers stems from its high structural complexity, with many cycles, producing multiple scattering from the disordered graph. If complexity is reduced by adding spatial correlations, as in a periodic network, the degree of lasing control is strongly reduced (see Supplementary Fig. 11).

In conclusion, we have shown experimentally and numerically that network lasers inherently display a large spectral control, for over 90% of the top 50 modes, via the choice of the pump profile. The degree of control stems from the network complexity, and could be increased for further flexibility or decreased for improved resiliency. Further design of lasing networks may lead to improved spectral and directional control^27^ and could also be extended to other systems described by wave propagation on networks^18^. In addition, network lasers are naturally adapted for on-chip integration, and could be made out of semiconductor materials to power next generation programmable light sources^7^, optical sensors^6^, and neuromorphic optical processors^8^.

## Methods

### Experiments on single mode lasing optimisation

The networks of dye-doped polymer nanofibers were electrospun as reported in Ref. 12, using a solution of polymethyl methacrylate (PMMA) and Rhodamine-6G (1% dye-to-polymer weight ratio) in a mixture of chloroform and dimethyl sulfoxide. Electrospinning was performed by loading the solution in a 1 mL syringe tipped with a metallic needle and applying a bias in the interval 10–15 kV between the needle and a Cu collector. The electrospun fibres have diameters in the range 200–500 nm, while the average length of the edges is of the order of few tens of micrometres. The density of fibers forming the networks mainly depends on the time interval of fibre deposition^28^, and it could be further controlled by using auxiliary metallic electrodes^29^. The networks were pumped using a *λ* = 532 nm pulsed laser (TEEM Microchip, pulse width 500 ps) and the emission was detected using an imaging spectrometer (Princeton Instruments Isoplane-320) with 1800 gr mm^−1^ holographic grating (0.05 nm resolution) and CCD camera (Princeton Instruments Pixis 400). Spectral measurements were taken with a narrow slit of 50 *μ*m width and the signal was integrated along the collection region. A digital micromirror device (DMD, Ajile AJD-4500) was used for beam shaping, resulting in a rectangular illumination spot of 300 × 480 *μ*m^2^ on the sample.

A derivative-free, greedy iterative algorithm was used to find the optimised pump patterns. Firstly, a coarse grid (8 × 5 grid with each pixel corresponding to 60 × 60 *μ*m^2^ size on sample) was used. Starting from the pixel closest to the centre of the grid, each pixel was switched off consecutively and the change in the intensity of the selected lasing mode was calculated using the recorded spectral counts. For a given lasing peak *p*, we calculated the following quality function at each optimisation step *n*:

1 $${{{\Phi }}}_{n}=\frac{{a}_{n}}{{a}_{n-1}}-1,\,{{{{{{{\rm{with}}}}}}}}\,{a}_{n}=\frac{{I}_{p}(n)}{\frac{1}{M}\mathop{\sum }\nolimits_{m=1;m\ne p}^{M}{I}_{m}(n)},$$

where *a*_*n*_ is the ratio of the intensity of the selected lasing peak *p* to the average intensity of the top *M* strongest lasing peaks (*M* = 10 in our experiments), all under pump pattern *n*. If Φ_*n*_ > 0, the pump on that pixel was kept off, otherwise it was switched back on, and the routine was iterated. The final pattern from a first run was then fed as the initial pattern for a subsequent re-run with a finer grid (patch sizes of 30 × 30 *μ*m^2^) for further optimisation.

### Numerical construction of Buffon graphs

Buffon graphs were generated by drawing lines on a plane at random points with random slope. The intersections of all the lines within a square region on the plane were obtained and the length of the line segments between intersections calculated. If a segment length was smaller than a minimum distance of 1 *μ*m, the intersection points were merged together to the median point. The final set of intersection points and line segments was then used to specify the graph vertices and adjacency matrix. The Buffon graphs used for numerical calculations were constructed to be similar to the polymer nanofiber networks, with 96 nodes, 131 edges, average degree 4, and mean edge length 23.8 *μ*m.

### Numerical model: SALT on networks (netSALT)

Lasers are usually described with two-level Maxwell-Bloch equations and numerically solved using finite difference methods^30^. An alternative, computationally efficient approach is to approximate these equations assuming stationarity of the population inversion and adopting the slowly-varying envelope approximation, resulting in the so-called SALT model^15,31^. The SALT model can be solved for arbitrary geometries, provided an efficient solver is available to compute the mode profiles in the lasing cavity.

Here, our cavity has the structure of a complex network, which we approximate as a quasi-1D system, where edges of the network are simple 1D cavities coupled via the nodes of the graph. This assumes that most of the light propagates in the direction of the edges, and that the complex scattering processes at the nodes can be well approximated with Neumann boundary conditions^32^. These two approximations are fundamental for what we call the netSALT model, i.e. SALT on networks. For full details on netSALT, see Supplementary Notes 1-3 and Supplementary Figs. 12 and 13, but we give here a summary.

The SALT equation for a one-dimensional cavity is

2 $${\partial }_{x}^{2}{u}_{\mu }+\left({\epsilon }_{ij}+\frac{{D}_{0}{\delta}_{{{{\mathrm{pump}}},}ij}{\gamma }_{\mu }}{1+{\sum }_{\nu }{I}_{\nu }{\Gamma }_{\nu }|{u}_{\nu }{|}^{2}}\right){k}_{\mu }^{2}{u}_{\mu }=0,$$

where *u*_*μ*_ is the normalised mode electric field and *δ*_pump,*ij*_ is the pump profile (equal to 1 on edges illuminated by the pump and 0 otherwise). The other parameters are: *ϵ*_*ij*_ the dielectric constant on each edge; *D*_0_ the pump strength; *γ*_*μ*_ = *γ*_⊥_/(*k*_*μ*_ − *k*_a_ + *iγ*_⊥_) is a function that defines the gain spectrum with centre at *k*_a_ and width *γ*_⊥_; and ${\Gamma }_{\mu }=-{{{{{{{\rm{Im}}}}}}}}({\gamma }_{\mu })$ is the Lorentzian gain curve. In our calculations we used *k*_a_ = 10.68 *μ*m^−1^ and *γ*_⊥_ = 0.5 *μ*m^−1^. The electric field (*u*_*μ*_) and pump strength (*D*_0_) in SALT equations are dimensionless and can be converted to physical units of electric field and inversion density, using ${E}_{\mu }={u}_{\mu }(\hslash \sqrt{{\gamma }_{\perp }^{\prime}{\gamma }_{\parallel }})/2g$ Vm^−1^ and $D={D}_{0}({\epsilon }_{0}\hslash {\gamma }_{\perp }^{\prime})/{g}^{2}$ cm^−3^, where *g* is the dipole moment matrix element (units Cm^−1^), *γ*_∥_ is the gain inversion rate and ${\gamma }_{\perp }^{\prime}=c{\gamma }_{\perp }$ the polarisation dephasing rate (units s^−1^)^15^.

To solve this equation, one needs the boundary conditions for each edge matched at each node of the underlying network. We use the theory of quantum graphs to derive a matrix equation for the electric field at the node. For each edge, we have *η*_*ij*_(*x*) obeying

3 $${\partial }_{x}^{2}{\eta }_{ij}(x)+{({n}_{ij}k)}^{2}{\eta }_{ij}(x)=0\qquad \forall \,(ij),$$

where *n*_*ij*_ is the index of refraction of the edge (*ij*). This has solutions of the form

4 $${\eta }_{ij}(x)={\lambda }_{ij}^{+}{e}^{ik{n}_{ij}x}+{\lambda }_{ij}^{-}{e}^{ik{n}_{ij}({l}_{ij}-x)},$$

where ${\lambda }_{ij}^{\pm }$ are the wave amplitudes, one-to-one with the wave amplitude *η*_*i*_ at node *i*. One can recast the boundary conditions at the nodes into a matrix *L*(*k*) (see Supplementary Note 1), such that the passive modes with wavenumber *k*_*μ*_ satisfy

5 $$L({k}_{\mu })\,{{{{{{{\boldsymbol{\eta }}}}}}}}=0,$$

where ***η*** is the vector containing the node wave amplitudes *η*_*i*_ as components.

The wave equation Equation 2 (see Supplementary Note 1) with nonlinear coupling between modes cannot be solved directly, but we obtain an approximation in several steps. First, we search for passive modes (without pump), i.e. with *D*_0_ = 0. These modes have a complex wavenumber *k*_*μ*_, whose imaginary part is related to the loss of the mode via the standard *Q*-factor

6 $${Q}_{\mu }=\frac{{{{{{{{\rm{Real}}}}}}}}({k}_{\mu })}{2\,|{{{{{{{\rm{Im}}}}}}}}({k}_{\mu })|}.$$

For each mode, we then search for the pump power *D*_0,*μ*_ for which ${{{{{{{\rm{Im}}}}}}}}({k}_{\mu }({D}_{0,\mu }))=0$ where *k*_*μ*_(*D*_0_) solves Equation 2 without the denominator in the nonlinear term. The wavenumber obtained is one of the so-called threshold lasing mode denoted here *u*_*μ*_. We then assume that above lasing, these modes do not change their profile significantly, thus the nonlinear coupling between the lasing modes due to the spatial hole burning term can be approximated by a matrix equation (see Supplementary Note 1). The lasing modes obtained through the approximated solution to Equation 2 are then given as *I*_*μ*_*u*_*μ*_, where *I*_*μ*_ is the mode intensity computed from this matrix equation.

### Numerical individual mode lasing optimisation

To numerically optimise a pump profile to single lase a specific mode, we would ideally maximise the ratio of the modal intensity of the target mode over the largest next lasing mode. However, as this quantity is numerically expensive to compute (due to the need to track modes in the complex plane), we approximate it using the overlapping factor Supplementary Equation 14, as an indication of the change of lasing threshold, and write the optimisation as a linear program (see Supplementary Note 1).

## Supplementary information

Supplementary Information

## Data Availability

The data used in this study is available on Figshare: 10.6084/m9.figshare.20506554.

## Code availability

Full code is available on Zenodo^33^, pypi https://pypi.org/project/netsalt/ and Github: https://github.com/arnaudon/netSALT.
