# Supplementary material for: Sensitivity and spectral control of network lasers
Source: Nat Commun. 2022 Oct 30;13:6493. doi: 10.1038/s41467-022-34073-3 (PMC9618558; doi:10.1038/s41467-022-34073-3)
Supplement: Supplementary file 1 — Supplementary Information [file 41467_2022_34073_MOESM1_ESM.pdf]

Supplementary Information:

Sensitivity and spectral control of network lasers

Dhruv Saxena,* Alexis Arnaudon,* Oscar Cipolato, Michele Gaio, Alain Quentel, Sophia Yaliraki, Dario Pisignano, Andrea Camposeo,† Mauricio Barahona,† and Riccardo Sapienza†

* These authors contributed equally to this work.

† andrea.camposeo@cnr.it, m.barahona@imperial.ac.uk, r.sapienza@imperial.ac.uk

Supplementary Note 1: NetSALT: extending SALT theory to lasing networks

Here we provide a more detailed derivation of netSALT, the numerical model used in the simulations of lasing networks presented in this work. The accompanying code is available at https://github.com/arnaudon/netSALT.

A. Open quantum graphs

A quantum graph is a metric graph (i.e., a graph where each edge (ij) has an associated length l_{ij} with an accompanying real length variable $x \in [0, l_{ij}])$ such that a function $\eta(x)$, which is defined on each edge and thus defined on the entire graph, satisfies a differential equation. In this case, we consider a quantum graph where the function $\eta(x)$ satisfies the Helmholtz differential equation

$$\partial_x^2 \eta_{ij}(x) + (n_{ij}k)^2 \eta_{ij}(x) = 0 \qquad \forall\,(ij)\,, \tag{1}$$

where the complex-valued $n_{ij} \in \mathbb{C}$ correspond to the index of refraction of the edge (ij). This equation being linear, it has solutions of the form

$$\eta_{ij}(x) = \lambda_{ij}^{+} e^{ikn_{ij}x} + \lambda_{ij}^{-} e^{ikn_{ij}(l_{ij}-x)}\,, \tag{2}$$

where the complex-valued $\lambda_{ij}^{\pm} \in \mathbb{C}$ represent the left- and right-propagating wave amplitudes. The continuity of $\eta(x)$ at each node is ensured by considering the edge function $\eta_{ij}(x)$ evaluated at the nodes:

$$\eta_i := \eta_{ij}(0) \quad \text{and} \quad \eta_j := \eta_{ij}(l_{ij}). \tag{3}$$

Then the conservation of energy at each node i can be shown to be equivalent to

$$(L(k)\,\boldsymbol{\eta})_i = \sum_{j\sim i} n_{ij} \frac{\eta_i \left(e^{ikn_{ij}l_{ij}} + e^{-ikn_{ij}l_{ij}}\right) - 2\eta_j}{e^{ikn_{ij}l_{ij}} - e^{-ikn_{ij}l_{ij}}} = 0 \qquad \forall i, \tag{4}$$

where the sum is over the nodes adjacent to i, and the matrix $L(k)$, dependent on the wavenumber, is a matrix acting on the node vector $\boldsymbol{\eta}$ with components η_i defined in (3). We refer to [1, 2] for more details.

The matrix $L(k)$ can be expressed in terms of an extension of the graph incidence matrix, which allows the simplification of the calculations of various quantities (see Equation (27)

and [3]). The condition (4) corresponds to an eigenvalue problem

$$L(k)\,\boldsymbol{\eta} = \mathbf{0}, \tag{5}$$

so, equivalently one can solve the corresponding scalar equation

$$\det\left(L(k)\right) = 0\,, \tag{6}$$

for discrete wavenumbers k_μ. Numerically, we solve this equation using the smallest eigenvalue of $L(k)$, which is efficient to compute with sparse matrices. Notice that the node representation contains a denominator term that diverges when $kn_{ij}l_{ij} \to n\pi$, with $n = 1, 2, \ldots$. This can cause numerical instabilities in rare cases, only encountered so far for graphs with several edges with the same length. We numerically fix it by adding a small noise to the edge lengths (or node positions).

Edges with one open end (i.e., a node of degree 1) are considered to be outside of the cavity and admit no incoming wave. This can be simply written as a projection of the matrix L, where elements corresponding to the outgoing waves are projected out, thus allowing them to take any value (and not enforced to be vanishing from the right hand side of (4)). This condition makes the quantum graph open, or lossy, and any solution of (4) must have a complex wavenumber k_μ. Henceforth, we make the distinction between *inner edges*, corresponding to the lasing cavity, and *outer edges*, corresponding to the open boundary of the cavity, also known as the last scattering surface in laser theory. For example, to describe the integration over the inner edges of the cavity, we use the shorthand notation

$$\int_{\mathrm{in}} dx = \sum_{(ij)\in\mathrm{in}} \int_0^{l_{ij}} dx. \tag{7}$$

For each passive mode k_μ, the standard $\mathcal{Q}$-factor is given by

$$\mathcal{Q}_\mu = \frac{\mathrm{Real}(k_\mu)}{2\,|\mathrm{Im}(k_\mu)|}\,. \tag{8}$$

B. The SALT equation on network edges

The SALT equation [4] describes the interaction of lasing modes under non-uniform pumping. In our formulation, the pump is naturally described by an amplitude D_0 and an edge indicator vector, denoted δ_{pump}, with components $\delta_{\mathrm{pump},ij} = 1$ if the pump is illuminating

edge (ij) and $\delta_{\text{pump},ij} = 0$ otherwise. We will also use the notation $\delta_{\text{pump}}(x)$ for the pump density, where $\delta_{\text{pump}}(x) = \delta_{\text{pump},ij}/l_{ij}$ for x on edge (ij).

The lasing modes are the modes with $\text{Im}(k_\mu) = 0$, and have the form

$$\Phi_\mu(x) = \sqrt{I_\mu}\, u_\mu(x),$$

where I_μ is the modal intensity, and u_μ is the mode profile, normalised as

$$\int_{\text{in}} \delta_{\text{pump}}\, u_\mu^2 dx = \sum_{(ij)} \delta_{\text{pump},ij}\, u_{\mu,ij}^2 = 1. \tag{9}$$

On edge (ij), the SALT equation [4] is a nonlinear extension of the Helmholtz equation (1) given by

$$\partial_x^2 u_{\mu,ij} + \left(n_{ij}^2 + D_0\, \delta_{\text{pump},ij} \frac{\gamma_\mu}{1 + \sum_\nu I_\nu \Gamma_\nu |u_{\nu,ij}|^2} \right) k_\mu^2 u_{\mu,ij} = 0\,, \tag{10}$$

where $\gamma_\mu = \frac{\gamma_\perp}{k_\mu - k_a + i\gamma_\perp}$ is a function that defines the gain spectrum with centre at k_a and width $\gamma_\perp$; and $\Gamma_\mu = -\text{Im}(\gamma_\mu)$ is the Lorentzian gain curve.

We will not re-derive this equation here from several approximations of the Maxwell-Bloch equation, but refer to [4] for more details and only mention that key to the SALT model is the steady state assumption, or stationary inversion approximation, where the inversion population (denoted by $D(x,t)$ in [5]) is taken to be constant in time. We refer to [5–7] for more detailed studies on the validity and generalisations of this approximation.

C. Finding threshold lasing modes

Before computing the modal intensities I_μ, we need the threshold lasing modes, which are obtained as solutions of the linear equation

$$\partial_x^2 u_{\mu,ij} + \left(n_{ij}^2 + D_{\mu,\text{th}} \delta_{\text{pump}} \gamma_\mu \right) k_{\mu,\text{th}}^2 u_{\mu,ij} = 0 \quad \text{where} \quad \text{Im}(k_{\mu,\text{th}}) = 0\,. \tag{11}$$

This is an implicit equation for the lasing threshold $D_{\mu,\text{th}}$, the threshold wavenumber $k_{\mu,\text{th}}$, and the threshold lasing mode profile $u_{\mu,ij}$.

Solving this equation involves an iterative algorithm on the value of D_0 to reach the condition $\text{Im}(k_{\mu,\text{th}}) = 0$, where the secular equation (6) is solved at each step. When D_0 is updated, we use the so-called Brownian ratchet algorithm [8] to search for the corresponding $k_\mu(D_0)$. This algorithm proposes random moves in the complex plane of wavenumbers, and

accepts only the ones that decrease the smallest eigenvalue of $L(k)$, and stops the search when a certain threshold is reached. The size of the proposed moves is adjusted according to how far we expect the mode to have moved.

To speed up the search of threshold lasing modes, we first estimate the location of a mode with a different D_0 by assuming that the mode profiles do not change with the pump, i.e. $u_{\mu,ij}(D_0) = \eta_{\mu,ij}$. First, recall that $\eta_{\mu,ij}$ are the passive modes, i.e., the solution of

$$\partial_x^2 \eta_{\mu,ij} + n_{ij}^2 k_{\mu,0}^2 \eta_{\mu,ij} = 0\,. \tag{12}$$

Multiplying (11) by $\eta_{\mu,ij}$ and integrating over the cavity, we obtain

$$k_\mu(D_0) = \frac{k_\mu(0)}{\sqrt{1 + D_0 \gamma_\mu f_{\mu,\mathrm{pump}}}}\,, \tag{13}$$

where f_μ is the pump overlapping factor of mode μ, defined as

$$f_\mu(\delta_{\mathrm{pump}}) = \frac{\int_{\mathrm{in}} \delta_{\mathrm{pump}}\, \eta_{\mu,ij}^2 dx}{\int_{\mathrm{in}} n_{ij}^2\, \eta_{\mu,ij}^2 dx}\,. \tag{14}$$

To estimate the pump strength at threshold, we use $\mathrm{Im}(k_{\mu,\mathrm{th}}) = 0$ in (13) to get

$$D_{\mu,\mathrm{th}}(\delta_{\mathrm{pump}}) \approx -\frac{1}{\mathcal{Q}_\mu \Gamma_\mu \mathrm{Real}(f_\mu(\delta_{\mathrm{pump}}))}\,, \tag{15}$$

where we also used the fact that $\mathrm{Real}(\gamma_\mu)$ is small for high $\mathcal{Q}$ modes.

To obtain an estimate of the complex wavenumber for an updated pump power $D_0' = D_0 + \delta D_0$, we use, instead of (13), the equation

$$k_\mu(D_0') = k_\mu(D_0)\sqrt{\frac{1 + D_0 \gamma_\mu f_{\mu,\mathrm{pump}}}{1 + D_0' \gamma_\mu f_{\mu,\mathrm{pump}}}}\,, \tag{16}$$

where γ_μ is now evaluated at $k_\mu(D_0)$. This equation is obtained similarly to equation (13) by replacing the passive mode with a pumped mode.

To find the threshold lasing modes, we linearly increase D_0 with small steps using (16) as a starting point for the Brownian ratchet algorithm to find the next partially pumped mode until we reach $\mathrm{Im}(k) = 0$, and then use a binary search (together with Brownian ratchet) to locate the position of the lasing threshold $D_{\mu,\mathrm{th}}$.

D. Interacting modal intensities

Once the threshold lasing modes are found, we can estimate their modal intensities as a function of the pump power D_0. To do this, we assume that the mode profiles above

threshold are the same as the mode profiles at threshold, and the threshold wavenumbers $k_{\mu,\text{th}}$ remain the same above threshold. With this approximation, which corresponds to the single pole approximation of [4], we can estimate the modal intensities of each mode, given a pump profile δ_{pump} and a pump strength D_0, as follows.

From (10), and using the normalisation (9), we follow [4] to arrive at the matrix equation

$$\sum_{\nu} T_{\mu\nu} I_\nu = \frac{D_0}{D_{\mu,\text{th}}} - 1\,, \tag{17}$$

where the sum is over lasing modes only, and the interaction matrix T has elements

$$T_{\mu\nu} = \Gamma_\nu \,\text{Real}\left(\int_{\text{in}} |u_\nu|^2 u_\mu^2 \delta_{\text{pump}}(x) dx\right)\,, \tag{18}$$

where the use of the real part is an approximation, since the integral in (18) has a small complex part in general.

Given D_0, the modal intensities are then simply found as

$$I_\mu(D_0) = \sum_{\nu} T^{-1}_{\mu\nu} \left(\frac{D_0}{D_{\nu,\text{th}}} - 1\right)\,, \tag{19}$$

if the set of lasing modes (indexed as ν) are known. To find the lasing modes, we follow again Ref. [4], and first compute the interacting lasing thresholds $D_{\mu,\text{int}}$. For the first lasing mode, the interaction threshold will be the lasing threshold $D_{\mu,\text{th}}$, but for the next lasing modes, interaction with the currently lasing modes will increase this value, until it reaches ∞, and no more modes can lase, leading to the phenomenon known as *gain clamping*.

To compute the sequence of lasing threshold modes, we proceed as follows. Let us assume that we have N lasing modes, and we seek to compute the interacting threshold of the next mode, indexed μ_{N+1}. At $D_0 = D_{\mu_{N+1},\text{int}}$, the mode μ_{N+1} will not lase, so $I_{\mu_{N+1}} = 0$, which, after some manipulation, gives

$$D_{\mu_{N+1},\text{int}} = D_{\mu_{N+1},\text{th}} \left(1 + \sum_{i=1}^{N} T_{\mu_{N+1}\mu_i} I_{\mu_i}\left(D_{\mu_{N+1},\text{int}}\right)\right)\,, \tag{20}$$

an implicit equation for the interacting lasing threshold. Due to linearity, we can simply rearrange terms to get

$$D_{\mu_{N+1},\text{int}} = D_{\mu_{N+1},\text{th}} \frac{1 - \sum_{i=0,j=0}^{N} T_{\mu_{N+1}\mu_i} T^{-1}_{\mu_i\mu_j}}{1 - \sum_{i=0,j=0}^{N} \frac{D_{\mu_{N+1},\text{th}}}{D_{\mu_j,\text{th}}} T_{\mu_{N+1}\mu_i} T^{-1}_{\mu_i\mu_j}}\,. \tag{21}$$

The next lasing mode is then the mode μ_{N+1} with the smallest value of $D_{\mu_{N+1},\text{int}}$. At some point, the denominator will become negative, corresponding to the gain clamping regime, where all other modes are suppressed by currently lasing modes, see [4] for more details on this phenomenon. Sometimes a lasing mode can stop lasing, due to a negative slope in (19), in which case this mode is removed from the list of lasing modes and will not contribute to this equation in the search of the next lasing mode.

The solution of this equation thus provides the so-called LL curves, with modal intensities of all the modes as a function of the pump power D_0, given as piece-wise linear functions, as well as the possibility to approximate lasing spectra at a given pump power, if a lasing linewidth is added.

E. Pump optimisation in netSALT with linear programming

To numerically optimise the pump profile in netSALT, we do not evaluate the full modal intensities, as this would result in a costly and slow algorithm, due to the search of modal trajectories in the complex plane to reach threshold. Instead, we use the linear approximation of the lasing threshold (15), which depends on the pump overlapping factor $f_\mu(\delta_{\text{pump}})$ given by (14). This overlapping factor can be written as a scalar product as it is given by a sum over the inside edges:

$$f_\mu(\delta_{\text{pump}}) = \sum_{(ij)} \delta_{\text{pump},ij}\, f_{\mu,ij} = f_\mu^T \delta_{\text{pump}},$$

where the component $f_{\mu,ij}$ is the overlapping factor for a pump defined to be applied only to edge (ij). The optimal pump $\widehat{\delta}_{\text{pump},\mu}$ is then the result of a minimisation problem of the form

$$\widehat{\delta}^{\epsilon}_{\text{pump},\mu} = \arg\min_{\delta} \frac{\max_\nu a_\nu^T \delta + \epsilon}{a_\mu^T \delta}\,, \tag{22}$$

where $a_\nu := f_\nu \mathcal{Q}_\nu \Gamma_\nu$ and the hyper-parameter $\epsilon > 0$ is a regulariser to avoid small pump profiles. From this optimisation, we obtain a family of solutions with various coverages of the network surface area on the target mode profile.

The original optimisation (22) is combinatorial since the edge indicator vector δ is integer-valued. To make the optimisation more amenable, we relax the problem to search for a *real* edge vector x, where each component (corresponding to an edge) is real and bounded

$0 < x_{ij} < 1$ such that

$$\widehat{x}_\mu^\epsilon = \arg\min_x \frac{\max_\nu a_\nu^T x + \epsilon}{a_\mu^T x}. \tag{23}$$

To solve this relaxed problem, we rewrite it as the following linear program (LP):

$$\begin{aligned} &\min_{y,m,t} m + \epsilon t \\ &a_\nu^T y \leq m, \quad \forall \nu \\ &a_\mu^T y = 1 \\ 0 < &y_i < t, \quad i = 0, \dots, n \end{aligned} \tag{24}$$

where $m := \max_\nu a_\nu^T x$ and we have used the Charnes-Cooper transformation

$$y = \frac{x}{a_\mu^T x}, \qquad t = \frac{1}{a_\mu^T x}. \tag{25}$$

To solve the LP (24), we use the public python software PuLP available at https://github.com/coin-or/pulp.

From the continuous solution of the LP (23), we obtain a discrete pump vector, $\widehat{\delta}_\mu^*$, where $\widehat{\delta}_{\mu,ij}^* = 1$ if $x_{ij} > 0$, followed by further thresholding of edges that have a small impact on the cost. Such edges are thought of as 'noise' from the SALT approximation, which reduce the resulting modal suppression ratio.

The results of this optimisation on the Buffon graph are illustrated in Supplementary Figure 7.

To optimise a pump for multi-mode lasing (see Supplementary Figure 10) using this linear programming framework, we replace the denominator of the cost (22) by the sum over the $a_\mu \to \sum'_\mu a'_\mu$ of μ' mode we wish to lase together.

F. Comparison with mode matching optimisation

A simpler strategy to produce an optimised pump profile for single mode lasing would be to pump only edges that have a large electric field of the target mode.

We have applied this method of pump optimisation whereby we select edges with largest amplitudes of the target mode we want to single lase whilst minimising the cost function defined in (22) (taken with $\epsilon = 0$). This is followed by removal of edges that have a small impact on the cost. The results of this optimisation on the Buffon graph are illustrated

in Supplementary Figure 7, and shown to produce a small pump if a mode has only a few edges concentrating most of its electric field amplitude, or a large pump for highly delocalised modes. Overall, this simpler scheme of mode matching is outperformed by the pump obtained from optimising (23) with linear programming. Yet mode matching can sometimes can result in better suppression ratio when the linear approximation used in the LP approach is not representative enough of the modal amplitudes.

Supplementary Note 2: Classical laser geometries

We have validated our netSALT calculations on simple laser cavities.

A. 1D-cavity laser

We model a 1D-cavity laser with non-uniform index and non-uniform pump profile from [4], as shown in Supplementary Figure 12a. Optical feedback due to reflection at the two ends of the cavity ($x = 0$ and $x = 1$) is taken into account by adding edges with unit index of refraction to the line graph, and imposing open boundary conditions at the outer nodes (in red). The index of refraction is set to 1.5 on the left 1/4 of the cavity and set to 3 on the remaining inner edges. The pump is applied to the left half of the cavity (on inner edges shown in green).

The remaining panels in Supplementary Figure 12 reproduce the results in Ref. [4]. The mode profiles of the first lasing mode (Supplementary Figure 12b) match exactly with Fig. 3a of Ref. [4], and the modal intensities (Supplementary Figure 12c) match Fig. 6 of Ref. [4]. The threshold lasing frequencies and the non-interacting lasing thresholds (not shown) also match the values reported in [4]. The code for this example is available in the github repository (https://github.com/arnaudon/netSALT).

B. Ring laser

In a ring with real index n and length L, the modes lie on the real axis with $k_m = \frac{2m\pi}{nL}$, where m is a positive integer. To model a ring laser with a finite $\mathcal{Q}$-factor, we require complex refractive index $\tilde{n}$ on the edges. Let $\tilde{n} = n + i\kappa$. Then the modes are given by complex values:

$$\mathrm{Re}(k_m) = \frac{n}{n^2 + \kappa^2} \frac{2m\pi}{L}, \qquad \mathrm{Im}(k_m) = -\frac{\kappa}{n} \mathrm{Re}(k) \tag{26}$$

Loss is therefore defined via κ, or equivalently by the $\mathcal{Q}$-factor (8). Supplementary Figure 13 shows the netSALT calculation of a uniformly pumped micro-ring laser with cavity length 10 μm and refractive index $1.5 + 0.005i$. This example is also provided in the github repository (https://github.com/arnaudon/netSALT).

Supplementary Note 3: Additional calculations in netSALT

We collect here additional formulae of the netSALT model introduced above.

A. Matrix representation of $L(k)$

The quantum graph equation (5) can be written in terms of matrices with analogues in classical graph theory. Given a network with N nodes and E edges, the matrix $L(k)$ can be interpreted and rewritten as a *quantum graph weighted Laplacian* of the form

$$L(k)\,\boldsymbol{\eta} = B^T(k)W^{-1}(k)B(k)\,\boldsymbol{\eta}\,, \tag{27}$$

where the matrix $B(k)_{N\times 2E}$ is an extension of the standard incidence matrix in graph theory with elements

$$B_{i,ij} = -1$$
$$B_{j,ij} = e^{ikl_{ij}}\,,$$

which contains both edge directions, and the diagonal weight matrix $W(k)_{2E\times 2E}$ is defined as

$$W_{ij,ij} = e^{2ikl_{ij}} - 1\,,$$

and the same for $W_{ji,ji}$. We refer to [3] for the details of the derivation of these equations.

B. Pump overlapping factor

The pump overlapping factor defined in (14) is explicitly given as

$$f_\mu = \sum_{(ij)} \delta_{\text{pump},ij} \int \eta_{\mu,ij}(x)^2 dx = \boldsymbol{\eta}^T B^T W^{-1} Z W^{-1} B\,\text{diag}(\delta_{\text{pump}})\,\boldsymbol{\eta}\,,$$

where the block-diagonal matrix Z is formed by the blocks

$$Z_{ij,ij} = \begin{pmatrix} \frac{e^{2ikl_{ij}}-1}{2ik} & l_{ij}e^{ikl_{ij}} \\ l_{ij}e^{ikl_{ij}} & \frac{e^{2ikl_{ij}}-1}{2ik} \end{pmatrix}.$$

C. Mode competition matrix

To simplify notation in this calculation, we consider k_μ to be complex and incorporate the index of refraction and the pump term into γ, and we drop edge indices ij in the expressions (28) below.

The T matrix defined in (18) has elements

$$T_{\mu\nu} = \Gamma_\nu \text{Real}\left(\int_{\text{in}} |u_\nu|^2 u_\mu^2 \delta_{\text{pump}}(x)dx\right) ,$$

and the integral can be written as

$$\int_{\text{in}} |u_\nu|^2 u_\mu^2 \delta_{\text{pump}}(x)dx = \sum_{(ij)} \delta_{\text{pump},ij} \begin{pmatrix} |\lambda_\nu^+|^2 \\ \lambda_\nu^+ \overline{\lambda_\nu^-} \\ \overline{\lambda_\nu^+} \lambda_\nu^- \\ |\lambda_\nu^-|^2 \end{pmatrix}^T \begin{pmatrix} A & E & E & B \\ C & F & F & D \\ D & F & F & C \\ B & E & E & A \end{pmatrix} \begin{pmatrix} (\lambda_\mu^+)^2 \\ \lambda_\mu^+ \lambda_\mu^- \\ \lambda_\mu^+ \lambda_\mu^- \\ (\lambda_\mu^-)^2 \end{pmatrix}$$

where

$$\begin{aligned} A &= \frac{e^{i(k_\nu - \overline{k}_\nu + 2k_\mu)l} - 1}{i(k_\nu - \overline{k}_\nu + 2k_\mu)} \\ B &= e^{2ik_\mu l} \frac{e^{i(k_\nu - \overline{k}_\nu - 2k_\mu)l} - 1}{i(k_\nu - \overline{k}_\nu - 2k_\mu)} \\ C &= \frac{e^{i(k_\nu + 2k_\mu)l} - e^{-i\overline{k}_\nu l}}{i(k_\nu + \overline{k}_\nu + 2k_\mu)} \\ D &= \frac{e^{ik_\nu l} - e^{i(2k_\mu - \overline{k}_\nu)l}}{i(k_\nu + \overline{k}_\nu - 2k_\mu)} \\ E &= e^{ik_\mu l} \frac{e^{i(k_\nu - \overline{k}_\nu)l} - 1}{i(k_\nu - \overline{k}_\nu)} \\ F &= e^{ik_\mu l} \frac{e^{ik_\nu l} - e^{-i\overline{k}_\nu l}}{i(k_\nu + \overline{k}_\nu)} . \end{aligned} \tag{28}$$

D. Calculation of the edge mean of $|E|^2$

The mode solution has the form (2):

$$\eta_{ij}(x) = \lambda_{ij}^+ e^{ikn_{ij}x} + \lambda_{ij}^- e^{ikn_{ij}(l_{ij}-x)}.$$

For brevity, we remove edge subscripts and take the modulus squared:

$$\eta(x)\overline{\eta(x)} = \lambda^+\overline{\lambda^+}e^{(ikn+\overline{ikn})x} + \lambda^+\overline{\lambda^-}e^{\overline{iknl}}e^{(ikn-\overline{ikn})x} + \lambda^-\overline{\lambda^+}e^{iknl}e^{(\overline{ikn}-ikn)x} + \lambda^-\overline{\lambda^-}e^{(ikn+\overline{ikn})(l-x)}$$

If we integrate from $x = 0$ to l, we get

$$\langle|E|^2\rangle = \frac{1}{l}\int \eta(x)\overline{\eta(x)}dx = \frac{1}{l}\lambda^+\overline{\lambda^+}\frac{e^{(ikn+\overline{ikn})l}-1}{ikn+\overline{ikn}} + \frac{1}{l}\lambda^+\overline{\lambda^-}\frac{e^{iknl}-e^{\overline{iknl}}}{ikn-\overline{ikn}} + \frac{1}{l}\lambda^-\overline{\lambda^+}\frac{e^{\overline{iknl}}-e^{iknl}}{\overline{ikn}-ikn} + \frac{1}{l}\lambda^-\overline{\lambda^-}\frac{e^{(ikn+\overline{ikn})l}-1}{ikn+\overline{ikn}}$$

This can be expressed in matrix form as

$$\langle|E|^2\rangle = \frac{1}{l}\begin{pmatrix}\lambda^+ & \lambda^-\end{pmatrix}\begin{pmatrix}\frac{e^{(ikn+\overline{ikn})l}-1}{ikn+\overline{ikn}} & \frac{e^{iknl}-e^{\overline{iknl}}}{ikn-\overline{ikn}} \\ \frac{e^{iknl}-e^{\overline{iknl}}}{ikn-\overline{ikn}} & \frac{e^{(ikn+\overline{ikn})l}-1}{ikn+\overline{ikn}}\end{pmatrix}\begin{pmatrix}\overline{\lambda^+} \\ \overline{\lambda^-}\end{pmatrix}$$

which can be computed from the node solution. Here $\overline{x}$ stands for the complex conjugate of x.

E. Calculation of the Inverse Participation Ratio

The inverse participation ration (IPR) provides a measure for the mode spread over the graph, and is given by:

$$\mathrm{IPR}_\mu = L_{tot}\frac{\sum_{(ij)}\int_0^{l_{ij}}|E_\mu|^4dx}{\left(\sum_{(ij)}\int_0^{l_{ij}}|E_\mu|^2dx\right)^2}, \tag{29}$$

where L_{tot} is the total edge length. This formula can be evaluated analytically using the complex wave amplitudes on the edges and the analytical solution of the electric field on each edge.

Supplementary Figures

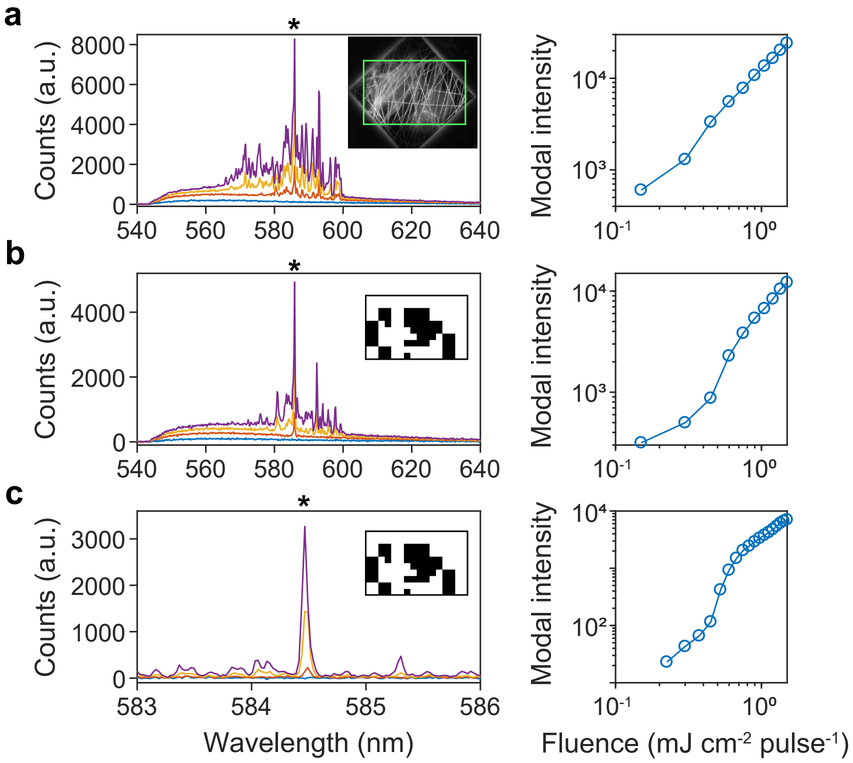

Supplementary Figure 1. Characterisation of lasing from polymer fiber networks. a Spectra with increasing pump fluence, at 0.1 (blue), 0.4 (red), 0.7 (yellow) and 1 mJ cm^{-2} pulse^{-1} (purple), when uniformly pumped, showing narrowing of emission and emergence of multiple modes above threshold. Inset shows fluorescence image of the network sample, with the area illuminated (300 × 480 μm^2) indicated by the green rectangle. Right panel shows the Light in-Light out (LL) curve of the strongest lasing mode (marked by *), plotted on log-log scale. Threshold behaviour (at ∼0.3 cm^{-2} pulse^{-1}) and characteristic lasing 'S' shape dependence of intensity with pump power is observed. **b** Same as in **a** but with a pump pattern optimised for the strongest lasing mode (pattern shown in inset). Reduction in number of modes lasing and improved side mode suppression are observed. Threshold with the non-uniform pump is slightly larger (∼0.5 cm^{-2} pulse^{-1}). **c** Same as in **b** but measured with a high resolution grating (see Methods). The dominant lasing mode has a linewidth of ∼0.05 nm.

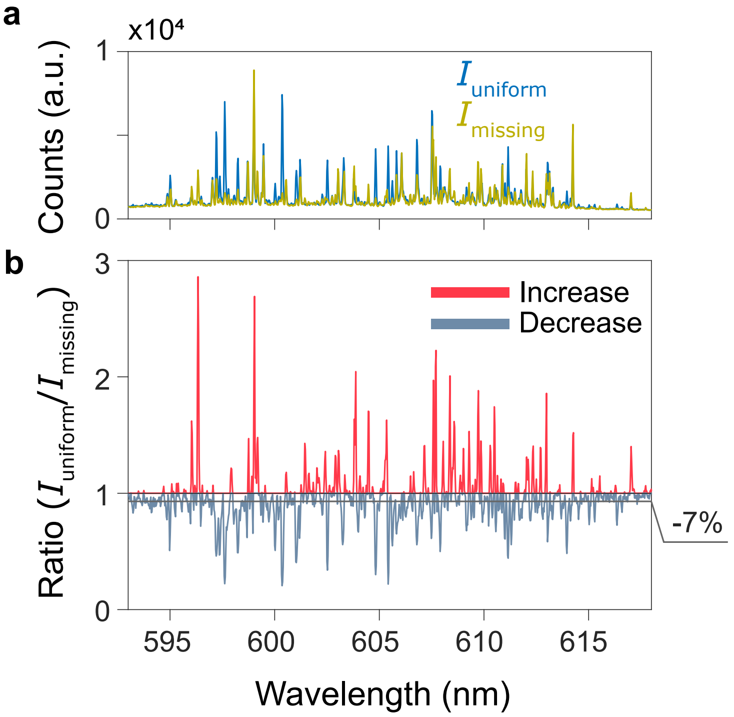

Supplementary Figure 2. **Sensitivity of network lasing spectrum.** **a** Experimental spectrum duplicated from Figure 1b of main text. **b** Ratio of intensities calculated from the spectra in **a**. When the pump pattern is modified by removing the pump from small central area (which results in a reduction of the delivered pump power by 7%), some lasing peaks increase by a factor 2.8 while others are attenuated down to 0.2 of the initial intensity.

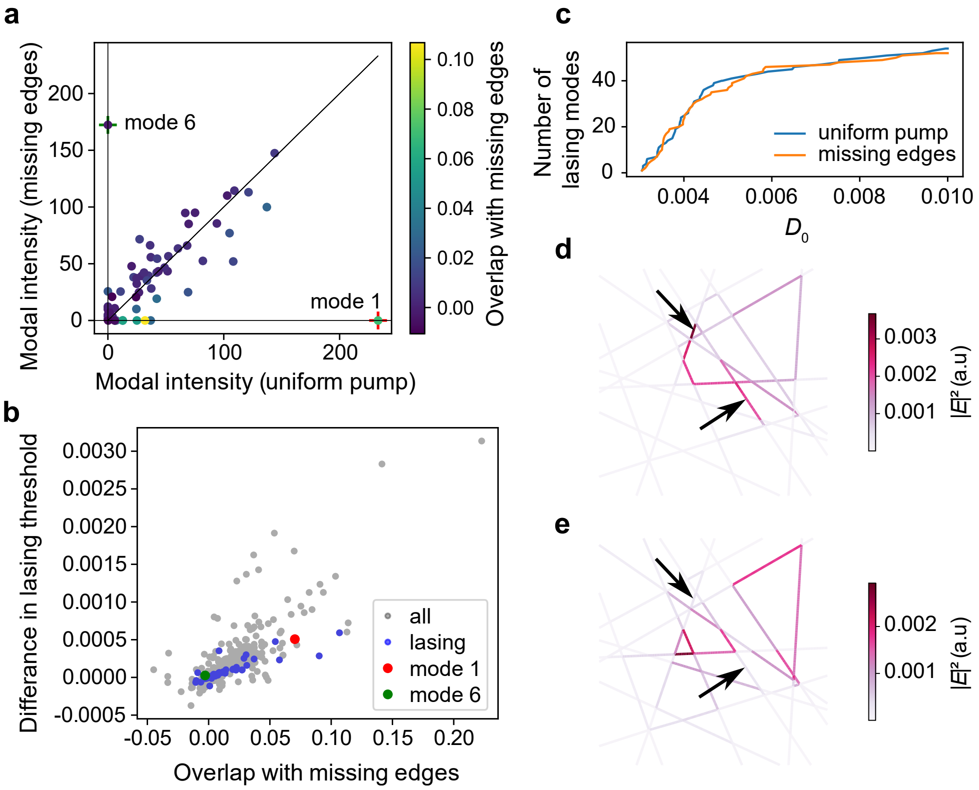

Supplementary Figure 3. **Missing edges experiment.** **a** We show the modal intensities at $D_0 = 0.01$ with uniform vs missing edge pump profiles, the colour corresponds to the overlap with 2 missing edges, computed as the real part of the f_μ factor with a pump localised on these two edges only. Modes with a large overlap with these edges are suppressed, such as the target mode 1 in red, and others appear, such as the mode 6 in green. **b** We show that overlap with missing edges (same quantity as the colour in panel **a** against the difference in lasing thresholds of all modes (grey), lasing modes (blue) and target mode 1 (red) and new mode 6 (green). We observe a strong correlation between these two quantities, indicating that the f_μ factor of each mode on edges is a good indicator of resulting changes in non-interacting lasing thresholds. **c** Number of lasing modes as a function of pump power for uniform and missing edges pump profiles. **d-e** We display the profile of modes 1 (in **d**) and 6 (in **e**) with missing edges indicated by arrows. We see a visual large overlap between these modes, but a small overlap of mode 6 with the missing edges (thus a small difference in lasing threshold, as shown in panel **b**.

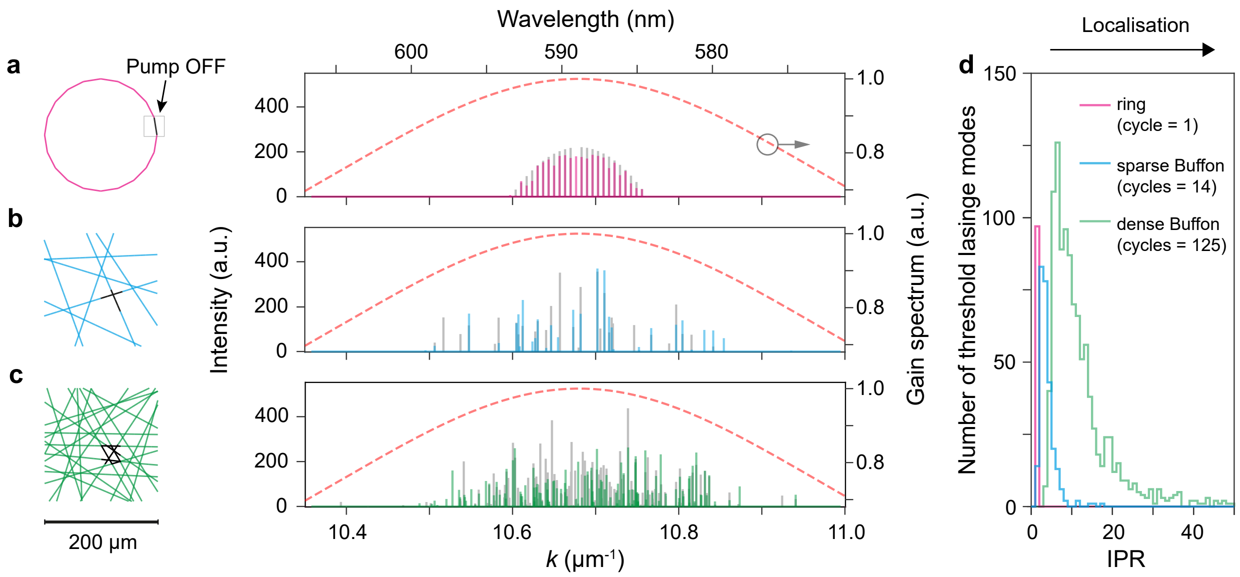

Supplementary Figure 4. Spectral sensitivity for different network density. a Pump profile (left) and lasing spectrum (right) for a single-loop network calculated with netSALT at pump power $D_0 = 0.02$. Pumped edges are in colour and un-pumped edges are in black. The total length of the un-pumped edges is 5% of the total graph length. Spectrum under uniform pumping (grey), also at $D_0 = 0.02$, is shown on the right panel for comparison. **b-c** The same calculations and plots are shown for two Buffon graphs with low and high edge density, respectively. The Buffon graphs clearly have higher spectral sensitivity compared to the single-loop graph. With increasing edge density, the total graph length increases corresponding to increase in the number of lasing modes, and larger spectral sensitivity. Density also affects the spatial extent of modes in the network, which in-turn has an effect on sensitivity as it changes the overlap between modes and the un-pumped edges. **d** Inverse participation ratio (IPR) distribution for the graphs in **a-c** shows that dense graphs (with more cycles) have larger fraction of localised modes and more number of competing lasing modes. The network lasers examined in the manuscript have edge densities between the low and dense Buffon graphs shown in **b** and **c**.

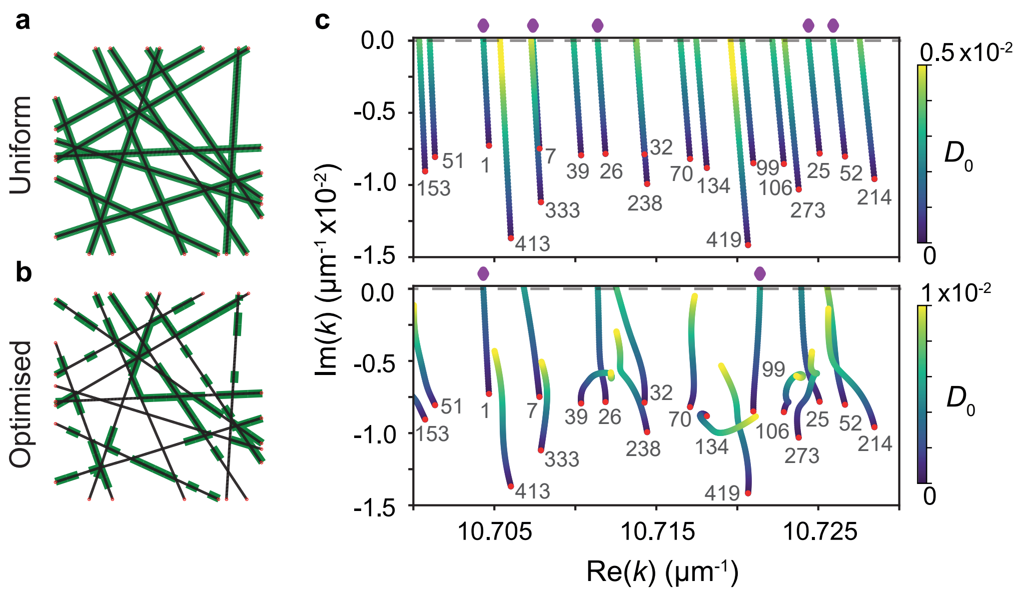

Supplementary Figure 5. **Mode trajectories under uniform and optimised pumping. a-b** Uniform pump profile and optimised pump profile for mode 1, as also shown in main manuscript Figure 2d insets. Dark green segments indicate region on edges that are pumped. **c** Mode trajectories in the complex k plane with the uniform and optimised pump profile (top and bottom panels, respectively) with increasing pump power D_0 (SALT units), values shown by colour bar. The trajectories are linear for all modes with uniform pumping. While gain in the network amplifies many modes to reach threshold $\mathrm{Im}(k) = 0$, only a subset of modes (indicated by purple diamonds) actually lase due to mode competition and gain saturation. The passive modes (without gain) are shown by red dots and modes are labelled in descending order of $\mathcal{Q}$-factor.

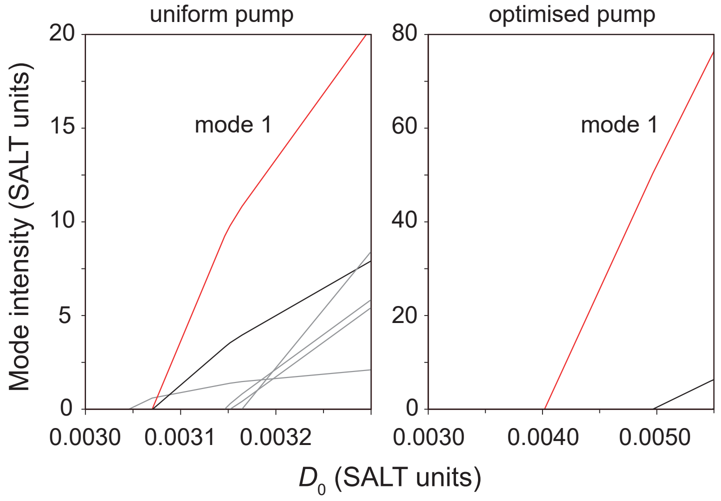

Supplementary Figure 6. Zoom in of LL curves shown in Figure 2c of main manuscript, where the lasing threshold of mode 1 becomes the smallest and the difference in threshold between mode 1 and the next lasing mode becomes large.

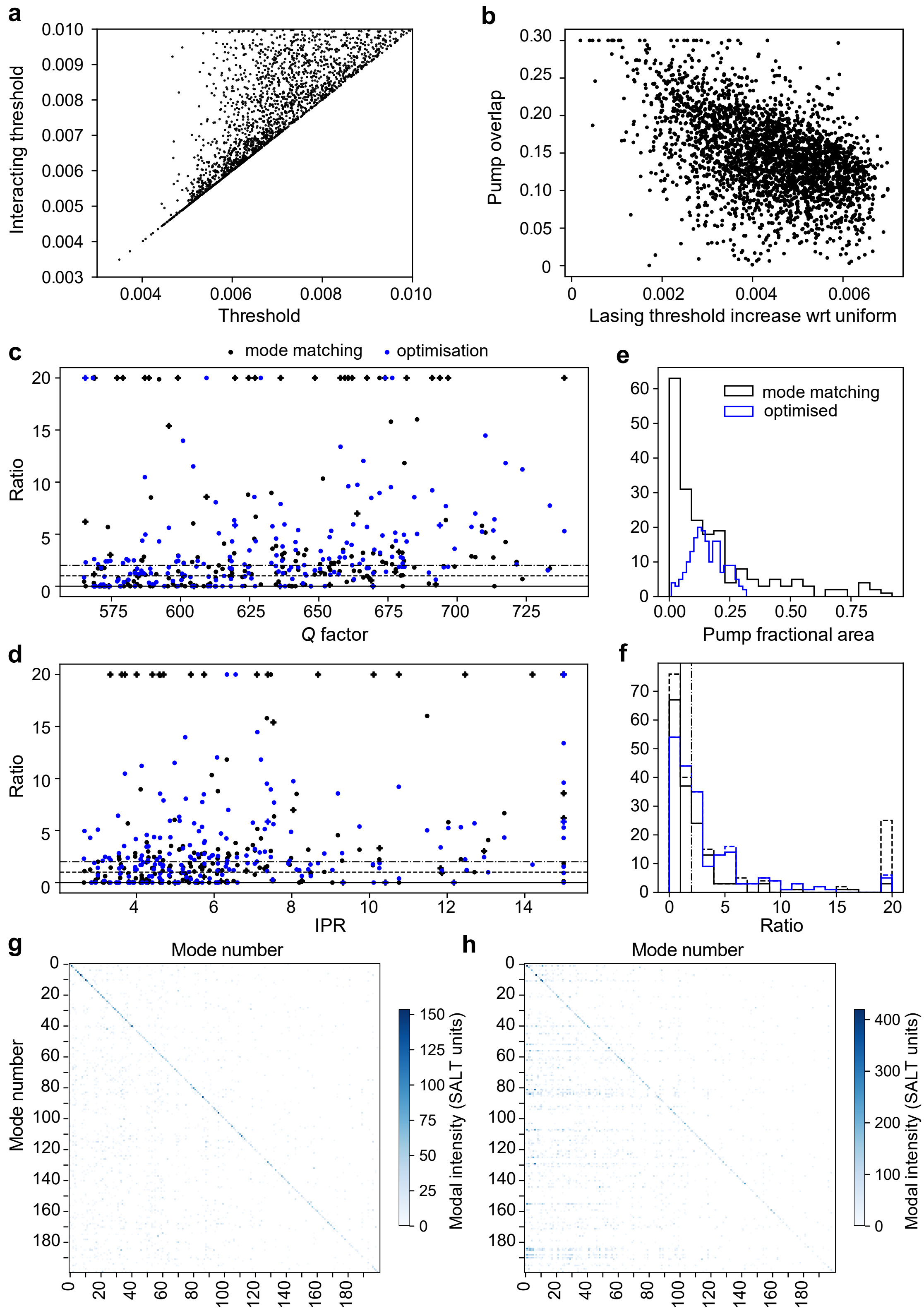

Supplementary Figure 7. **Mode control with optimisation and mode matching.** **a** Interacting lasing thresholds vs. lasing threshold, showing a large range of amount of modal interactions. (Cont'd next page.)

Supplementary Figure 7. (Cont'd.) **b** Overlap with pump (f_μ factor) vs. lasing threshold difference with respect to uniform pumping, showing that low lasing thresholds modes have large overlap with the pump (each dot is a mode for 20 optimisations). **c** Modal ratio vs $\mathcal{Q}$-factor for each of 100 optimisations (blue) and mode matching (black) pumps, at pump power $D_0 = 0.01$. Crosses are for pumps with small surface area (smaller than 2% of the surface area). The optimisation and mode matching were not limited in surface area profiles, and mode matching works well for small pumps on localised modes (with not other modes lasing) but is not realistic experimentally. Overall, the optimisation provides pumps with larger areas and better ratios. With optimisation, 66/100 have ratio larger than 2, and 90/100 larger than 1, while for mode matching, 39/100 have larger than 2 and 59/100 larger than one. **d** Same as **c**, but with IPR instead of $\mathcal{Q}$-factor, showing no dependence on mode localisation. **e** Fraction of pump area for optimisation and mode matching, showing that mode matching either under or over estimates the pump area. **f** Distribution of mode suppression ratio for all pumps in dashed, and large pumps (larger than 2% of surface area) in thick lines. **g** Full controllability matrix, where each row contains the modal intensities of a linear programming optimisation calculated at $D_0 = 0.01$. **h** Same as **g** but for mode matching optimisation, showing larger off-diagonal values, thus worse single lasing regimes than with the optimisation.

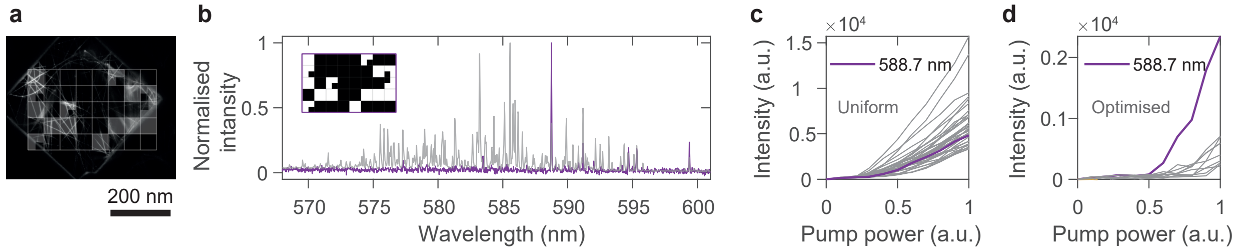

Supplementary Figure 8. **Optimisation of a high threshold mode.** Optimisation performed on the 11th strongest intensity mode from the uniform pumped spectrum. **a** Optical image of the sample when illuminated with the optimised pattern. Data is taken from the same area of the sample as in Figure 3 of the main text. **b** Normalised spectrum at full power with uniform pump (grey) and optimised pump pattern (purple). **c-d** Light in-light out (LL) curve showing intensity of lasing modes with pump power with uniform and optimised pump profile, respectively.

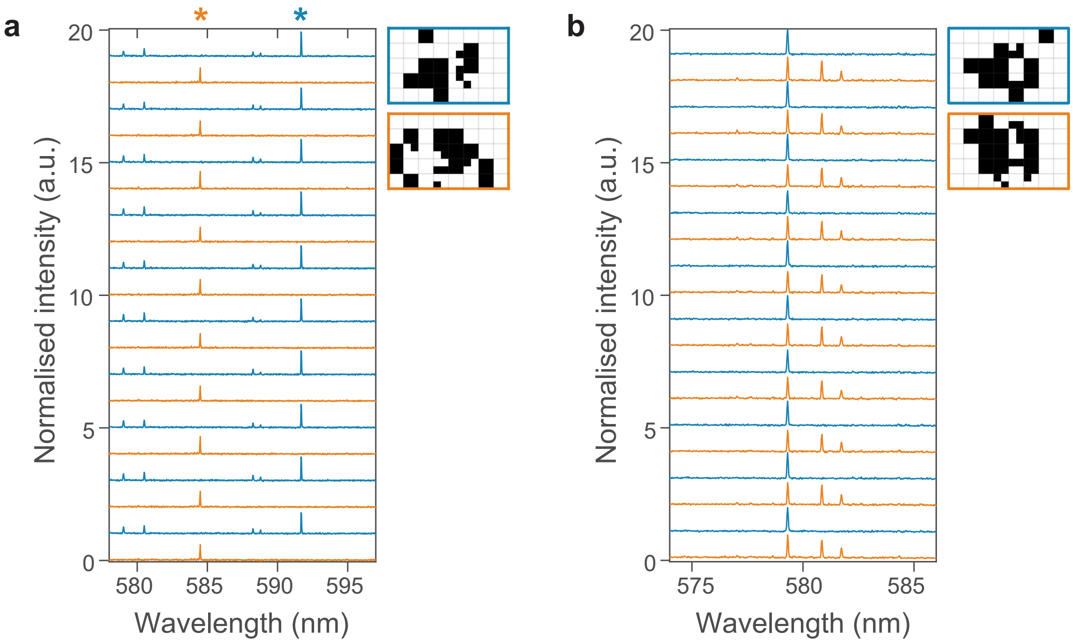

Supplementary Figure 9. **Spectral modulation using optimised pump patterns.** Lasing spectrum from the network laser is stable and can be illuminated with different optimised pump patterns to alternate between different lasing modes. An example of alternating 10 times with two single mode lasing spectra in **a** and between single mode and three mode lasing spectra in **b**. Respective pump patterns are shown on top right insets. Data in **a** and **b** were taken from a different region of the sample compared to Figure 3 of the main text.

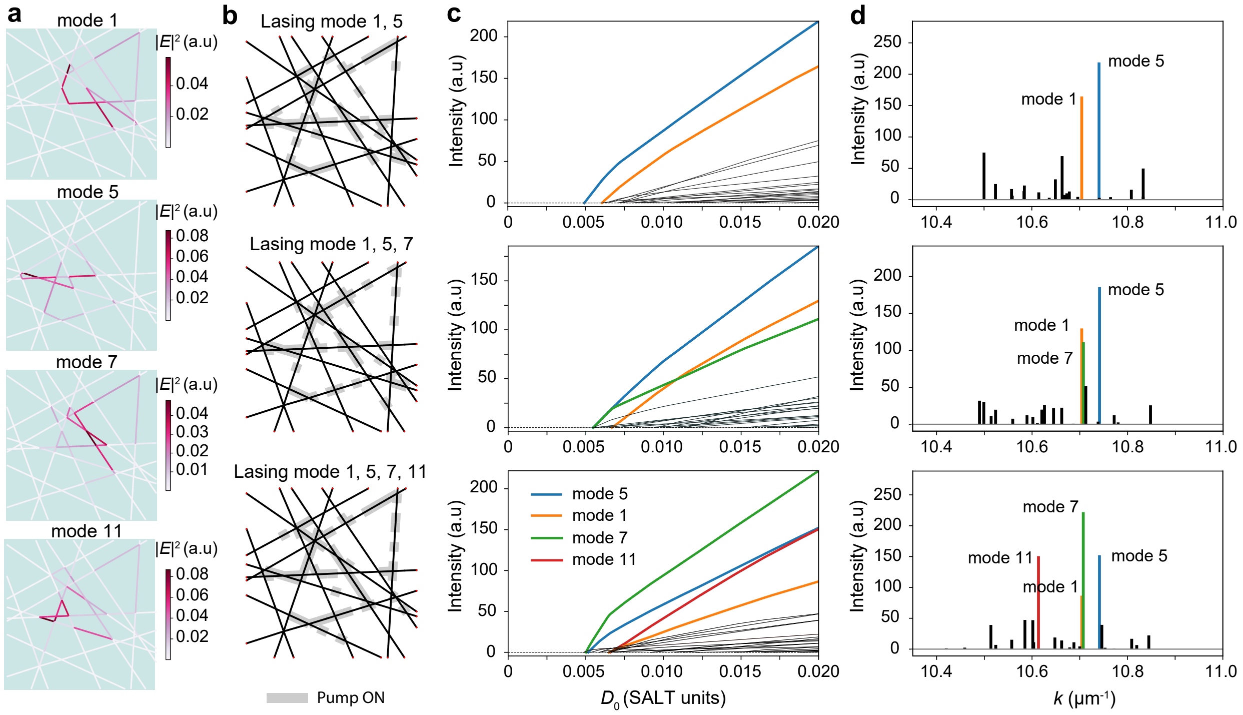

Supplementary Figure 10. **Multi-mode optimisation with netSALT. a** Mode profile of the four modes targeted to lase, where mode 1 and 7 have the most spatial overlap. **b** Optimised pump profiles to lase 2, 3 or 4 of these modes. **c** Modal intensities as a function of pump power for these three multi-mode optimisations, showing a faster increase of the targeted mode, despite the modal competitions. **d** Synthetic spectra of the three optimisations at $D_0 = 0.02$.

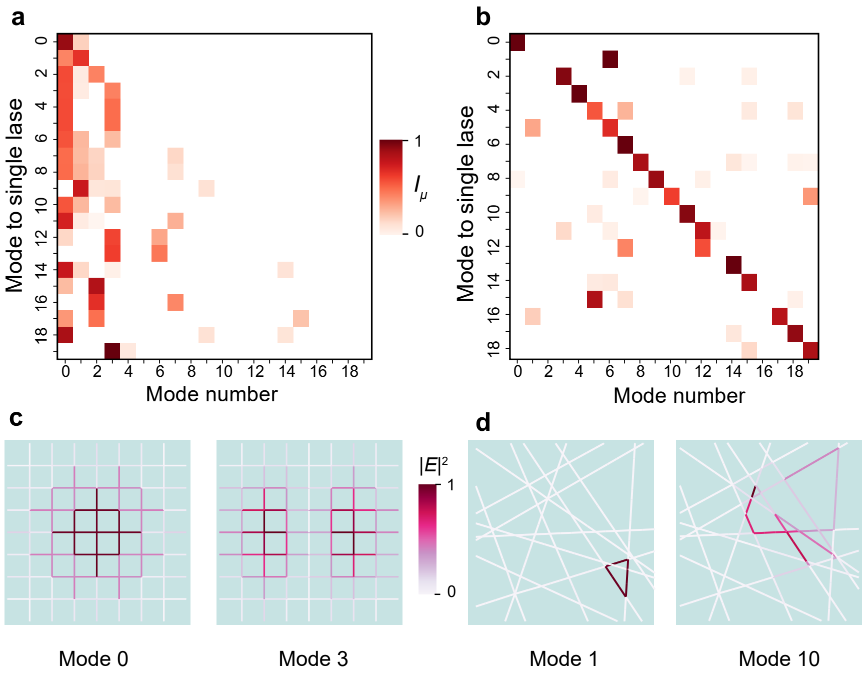

Supplementary Figure 11. **Periodic vs random network.** **a-b** Controllability map for a periodic network, **a**, in the form of a grid, which shows poor control of the lasing action compared to a similar size network with a random topology, **b**, which instead can be well controlled. The maps are calculated at the same pump power $D_0 = 0.01$. Two representative modes are shown in **c** and **d**, respectively.

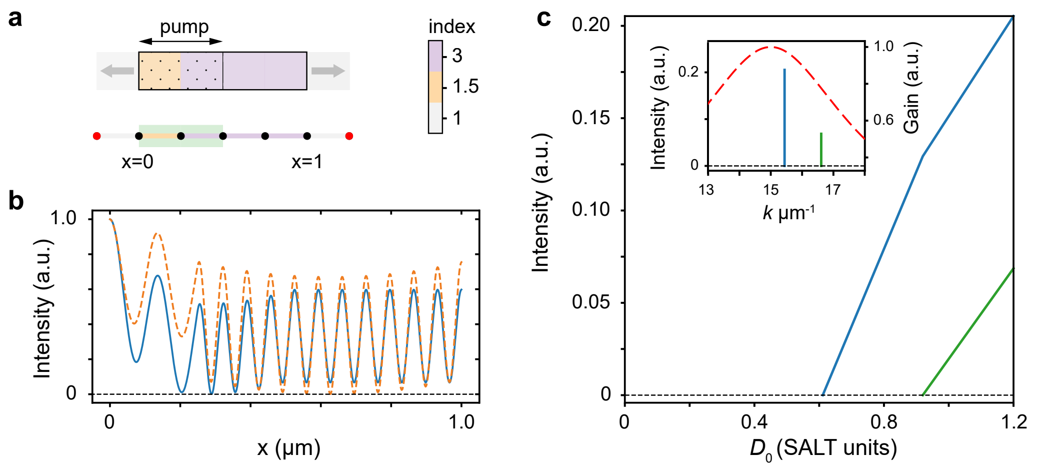

Supplementary Figure 12. **Example of 1D laser cavity.** **a** Schematic of 1D laser cavity with non-uniform index profile and non-uniform pump-profile. Graph representation of the example is shown below. **b** Normalised electric field intensity profile of the lasing mode at threshold (blue) and without pumping (orange dashed). **c** Lasing intensity as a function of pump intensity D_0 and spectrum at $D_0 = 1.2$ in the inset.

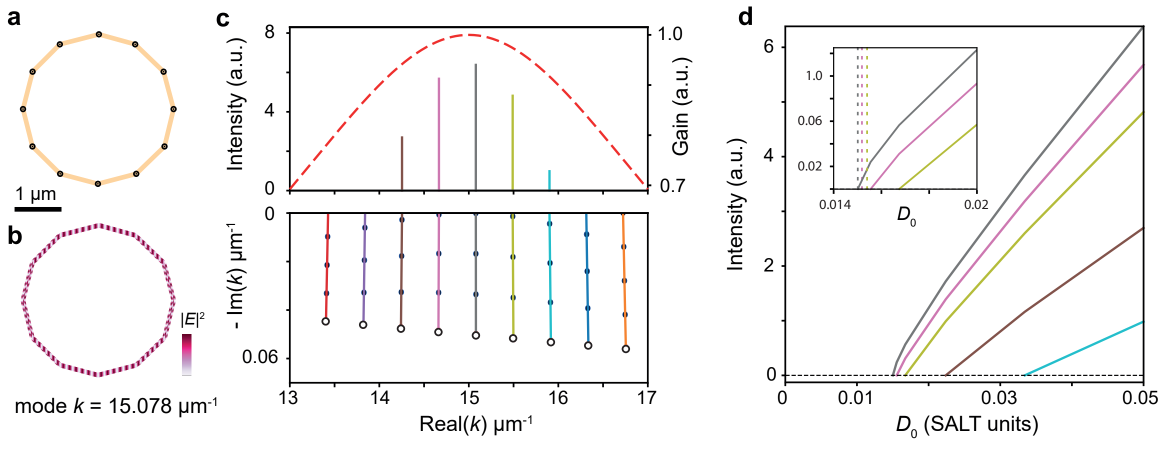

Supplementary Figure 13. **Example of a ring laser.** **a** Ring graph with $L = 10$ μm and uniform index $n = 1.5 + 0.005i$. **b** Electric field intensity profile of the lasing mode (axial order $m = 36$) at threshold $D_{0,\mathrm{th}} = 0.015$. **c** Lasing spectra at $D_0 = 0.05$ showing intensities of the 5 lasing modes. Gain spectrum (red dashed line) has the following parameters: $k_a = 15$ and $\gamma_\perp = 3$. Bottom panel shows the mode trajectories of the passive modes (open circles) at different D_0 values: $D_0 = 0.005$, 0.01, 0.015 (black circles). **d** LL curve for the ring laser. Inset shows zoomed in view of the first three lasing modes and their non-interacting thresholds (vertical dashed lines).

Supplementary References

[1] S. Gnutzmann and U. Smilansky, Advances in Physics **55**, 527 (2006).

[2] G. Berkolaiko and P. Kuchment, *Introduction to quantum graphs*, 186 (American Mathematical Soc., 2013).

[3] A. Arnaudon and M. Barahona, In preparation (2022).

[4] L. Ge, Y. Chong, and A. D. Stone, Physical Review A **82**, 063824 (2010).

[5] L. Ge, R. J. Tandy, A. D. Stone, and H. E. Türeci, Optics Express **16**, 16895 (2008).

[6] M. Liertzer, L. Ge, A. Cerjan, A. Stone, H. E. Türeci, and S. Rotter, Physical Review Letters **108**, 173901 (2012).

[7] S. Esterhazy, D. Liu, M. Liertzer, A. Cerjan, L. Ge, K. Makris, A. Stone, J. Melenk, S. Johnson, and S. Rotter, Physical Review A **90**, 023816 (2014).

[8] P. K. Newton and G. Chamoun, Proceedings of the Royal Society A: Mathematical, Physical and Engineering Sciences **463**, 1525 (2007).
